# FGF10/FGFR2b signaling plays essential roles during in vivo embryonic submandibular salivary gland morphogenesis

**DOI:** 10.1186/1471-213X-5-11

**Published:** 2005-06-22

**Authors:** Tina Jaskoll, George Abichaker, Daniel Witcher, Frederic G Sala, Saverio Bellusci, Mohammad K Hajihosseini, Michael Melnick

**Affiliations:** 1 Laboratory Developmental Genetics, University of Southern California, Los Angeles, CA, USA; 2 Department of Surgery, and Division of Developmental Biology, the Saban Research Institute of Children's Hospital Los Angeles, Los Angeles, CA, USA; 3 School of Biological Sciences, University of East Anglia (UEA), Norwich – Norfolk, UK

## Abstract

**Background:**

Analyses of *Fgf10 *and *Fgfr2b *mutant mice, as well as human studies, suggest that FGF10/FGFR2b signaling may play an essential, nonredundant role during embryonic SMG development. To address this question, we have analyzed the SMG phenotype in *Fgf10 *and *Fgfr2b *heterozygous and null mutant mice. In addition, although previous studies suggest that the FGF10/FGFR2b and FGF8/FGFR2c signaling pathways are functionally interrelated, little is known about the functional relationship between these two pathways during SMG development. We have designed *in vivo *and *in vitro *experiments to address this question.

**Results:**

We analyzed *Fgf10 *and *Fgfr2b *heterozygous mutant and null mice and demonstrate dose-dependent SMG phenotypic differences. Hypoplastic SMGs are seen in *Fgf10 *and *Fgfr2b *heterozygotes whereas SMG aplasia is seen in *Fgf10 *and *Fgfr2b *null embryos. Complementary *in vitro *studies further indicate that FGF10/FGFR2b signaling regulates SMG epithelial branching and cell proliferation. To delineate the functional relationship between the FGF10/FGFR2b and FGF8/FGFR2c pathways, we compared the SMG phenotype in *Fgfr2c*^+/*Δ*^*/Fgf10*^+/- ^double heterozygous mice to that seen in wildtype, *Fgf10*^+/- ^(*Fgfr2c*^+/+^*/Fgf10*^+/-^) and *Fgfr2c*^+/*Δ *^(*Fgfr2c*^+/*Δ*^*/Fgf10*^+/+^) single heterozygous mutant littermates and demonstrate genotype-specific SMG phenotypes. In addition, exogenous FGF8 was able to rescue the abnormal SMG phenotype associated with abrogated FGFR2b signaling *in vitro *and restore branching to normal levels.

**Conclusion:**

Our data indicates that FGF10/FGFR2b signaling is essential for the SMG epithelial branching and histodifferentiation, but not earliest initial bud formation. The functional presence of other endogenous signaling pathways could not prevent complete death of embryonic SMG cells in *Fgf10 *and *Fgfr2b *null mice. Though we were able to rescue the abnormal phenotype associated with reduced *in vitro *FGF10/FGFR2b signaling with exogenous FGF8 supplementation, our results indicate that the FGF10/FGFR2b and FGF8/FGFR2c are nonredundant signaling pathways essential for *in vivo *embryonic SMG development. What remains to be determined is the *in vivo *functional relationship between the FGF10/FGFR2b signal transduction pathway and other key signaling pathways, and how these pathways are integrated during embryonic SMG development to compose the functional epigenome.

## Background

Mouse submandibular gland (SMG) development is initiated with a thickening of the oral epithelium of the mandibular arch around embryonic day 11.5 (E11.5) and is best conceptualized in stages[1,2]. In the *PreBud *Stage, SMG development begins as thickening of the oral epithelium adjacent to the tongue. During the *Initial Bud *Stage, this thickening grows down into mandibular arch mesenchyme to form the initial SMG bud. With continued epithelial proliferation and downgrowth, the SMG primordium becomes a solid, elongated epithelial stalk terminating in a bulb. Repeated end-bud branching results in the formation of a network of epithelial branches and terminal buds (the *Pseudoglandular *Stage). These epithelial branches and terminal buds hollow out by cell apoptosis during the *Canalicular *and *Terminal Bud *Stages, respectively, to form the ductal system and presumptive acini, with mucin protein being produced by the presumptive acini.

Morphogenesis of complex organs such as the SMG is regulated by the functional integration of parallel and broadly related signaling pathways which regulate cell proliferation, apoptosis and histodifferentiation [3-6]. To understand the complex interactions within this dynamic signaling network, one must first determine the contribution of individual pathways and identify those which play essential, nonredundant roles during embryonic SMG initial bud formation, branching morphogenesis and histodifferentiation.

The FGF family, with at least 22 members, mediates diverse biological functions such as cell proliferation, branching morphogenesis and histodifferentiation by binding and activating four tyrosine kinase receptors (FGFR 1–4) [see reviews [7-9]]. Tissue-specific alternate splicing of the *Fgfr1*, *Fgfr2 *and *Fgfr3 *genes generates isoforms which differentially bind specific FGF ligands [8,10]. Ligand-receptor binding potentially activates multiple intracellular cascades, including the ERK/RAS/MAPK, P13K, and PLC-γ/PKC pathways [see reviews [8,9,11]].

Functional studies have demonstrated that embryonic SMG epithelial cell proliferation, branching morphogenesis, and histodifferentation are regulated through growth factor, cytokine, and transcription factor-mediated signaling pathways, including EGF, TGF-β, Shh, FGFs, and Eda [2,6,12-21]. Although FGFs have been implicated in embryonic SMG development [13,15,22-24], a more complete understanding of their precise roles will provide insight into the complex network of parallel and broadly-related signaling pathways which regulate SMG organogenesis.

Gene targeting studies have clearly shown the importance of the FGFR2b (FGFR2-IIIb) signaling pathway for embryonic morphogenesis [22,23,25]. *Fgfr2b*^-/- ^null mice die at birth due to lung insufficiency and exhibit severe dysmorphic organs, including agenesis or dysplasia of the lungs, mammary glands, pancreas, thyroid, teeth, and limbs [22,23,25,26]. Although FGF1, FGF3, FGF7, and FGF10 bind with high affinity to FGFR2b [8,10], the phenotypic similarities between *Fgf10 *and *Fgfr2b *null mice [22-25,27] indicate that FGF10 is the major ligand for FGFR2b *in vivo*. Of particular interest is the absence of SMGs in E14.5 and older *Fgfr2b *null mice and newborn *Fgf10 *null mice [23-25]. However, whether this glandular absence is due to the lack of development of a SMG initial bud (i.e. agenesis) or subsequent aplasia of the initial bud was heretofore unknown.

Recently, Entesarian et al. [28] have shown the importance of *FGF10 *gene dosage for salivary gland development in humans. Individuals with autosomal dominant ALSG (aplasia of lacrimal and salivary gland) exhibit hypoplastic or absent parotid and submandibular glands. ALSG was mapped to 5p13.2-5q13.1 to include the *FGF10 *gene; heterozygous *FGF10 *mutations were identified in all family members with ALSG. Complementary study of adult *Fgf10*^+/- ^mutant mice revealed that *Fgf10 *heterozygotes have absent parotid glands and smaller SMGs although other organs such as lungs, liver, spleen, pancreas, thyroid, limbs appeared normal.

Taken together, the literature suggests that FGF10/FGFR2b signaling plays an essential, nonredundant, dose-dependent role during embryonic SMG development. To address this postulate, we evaluated SMG development in *Fgfr2b *and *Fgf10 *heterozygous mutant and null mice and demonstrate dose-dependent differences in SMG phenotypes. In a complementary set of *in vitro *experiments, we confirm the importance of FGF10/FGFR2b signaling and demonstrate that enhanced FGF10/FGFR2b signaling significantly induces, and abrogated FGF10/FGFRb signaling significantly diminishes, SMG branching morphogenesis and cell proliferation.

Our previous analyses of mutant mice and functional *in vitro *studies indicate that the FGF8/FGFR2c signaling pathway is essential for embryonic SMG epithelial branching morphogenesis and histodifferentiation [2,13]. Little is known about the functional relationship between the FGF10/FGFR2b and FGF8/FGFR2c pathways during SMG development. Thus, we evaluated the SMG phenotype in *Fgfr2c*^+/*Δ*^*/Fgf10*^+/- ^double heterozygous mice, compared them to *Fgf10*^+/- ^(*Fgfr2c*^+/+^*/Fgf10*^+/-^) and *Fgfr2c*^+/*Δ *^(*Fgfr2c*^+/*Δ*^*/Fgf10*^+/+^) single heterozygous mutant, as well as wildtype (WT), littermates and demonstrate genotype-specific SMG phenotypes. Though we were able to rescue the abnormal phenotype associated with reduced *in vitro *FGF10/FGFR2b signaling with exogenous FGF8 peptide supplementation, our results indicate that the FGF10/FGFR2b and FGF8/FGFR2c are nonredundant signaling pathways essential for *in vivo *embryonic SMG development.

## Results

**Figure 1:**
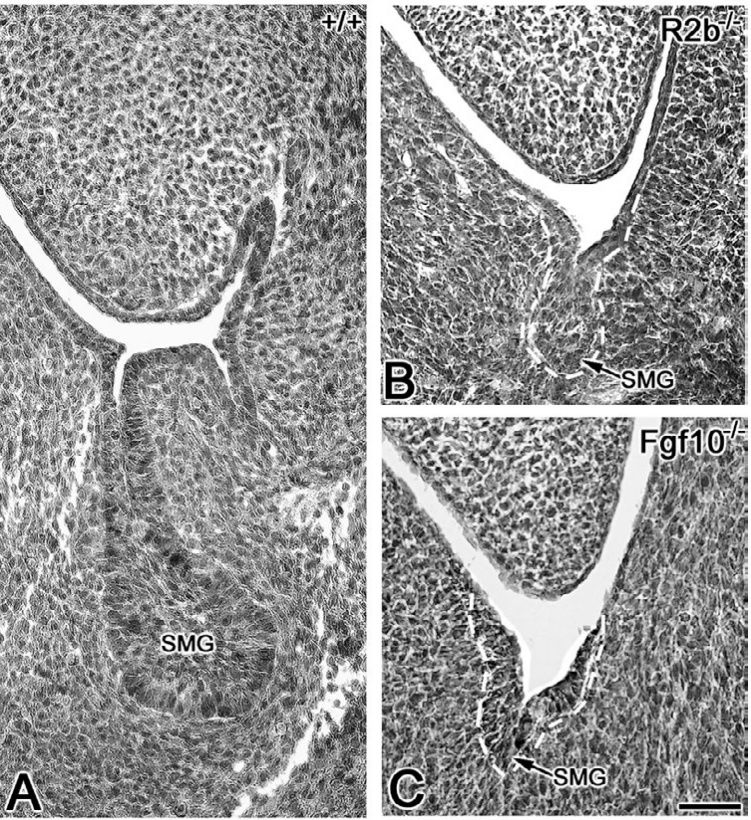
**Abnormal SMG phenotypes in E12.5 *Fgfr2b *and *Fgf10 *null mutants A**. E12.5 WT (+/+) mouse. B. E12.5 *Fgfr2b*^-/- ^(R2b^-/-^) null mouse. C. E12.5 *Fg10*^-/- ^null mouse. In the WT mouse (A), the *Initial Bud *Stage SMG is seen in the mandible ventrolateral to the tongue. By contrast, the SMG bud (outlined in white) in *Fgfr2b*^-/- ^(B) and *Fg10*^-/- ^(C) mice is extremely small, resembling the earliest *Initial Bud *Stage SMG. Bar, 50 μm.

**Figure 2:**
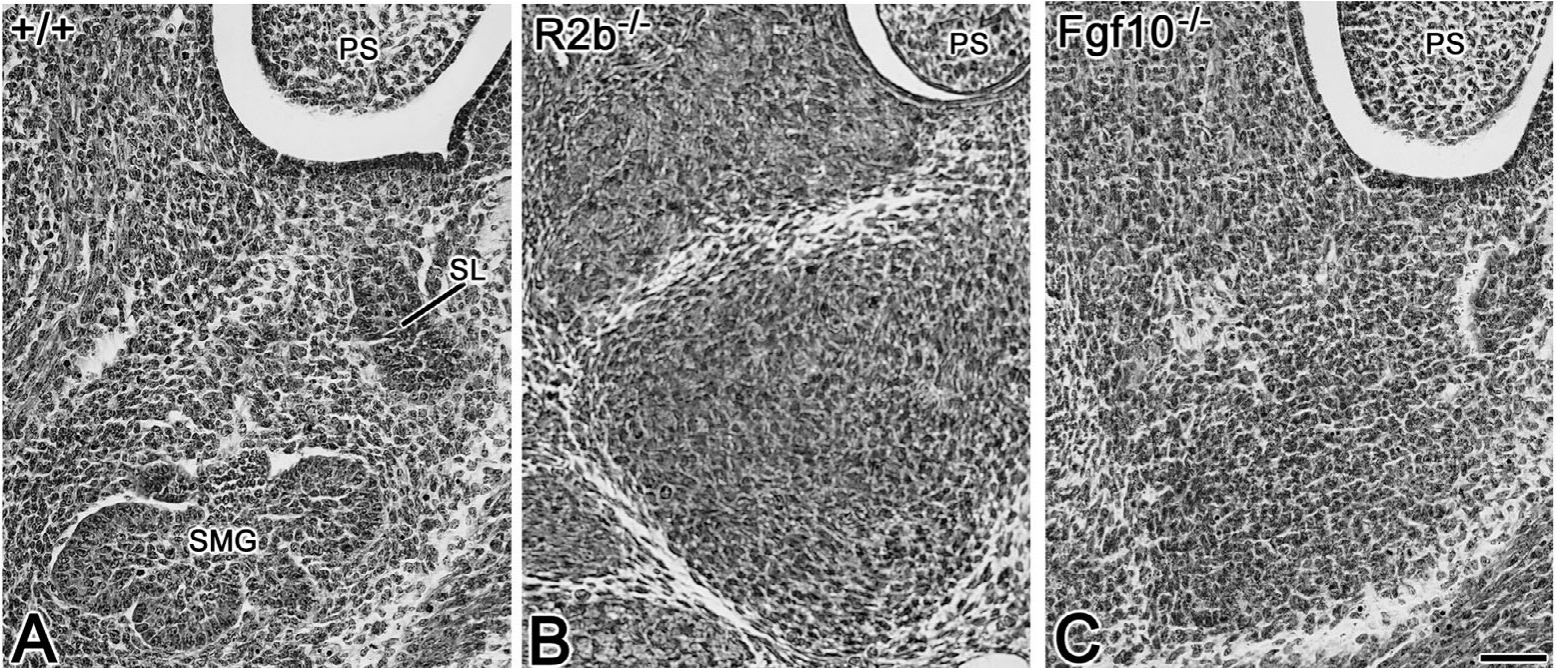
**SMG aplasia in *Fgfr2b *and *Fgf10 *null mice. **A. E13.5 WT mouse. B. E13.5 *Fgfr2b*^-/- ^null mouse. C. E13.5 *Fg10*^-/- ^null mouse. The E13.5 WT SMG (A) has achieved the *Late Initial Bud *Stage, with several branches being seen in the end-bulb epithelium; a small sublingual gland (SL) bud is seen lateral to the SMG bud. In *Fgfr2b*^-/- ^(B) and *Fg10*^-/- ^(C) embryos, no SMGs are found; undifferentiated mesenchyme is seen in the site normally occupied by SMG epithelia. PS-palatal shelf. Bar, 50 μm.

**Figure 3:**
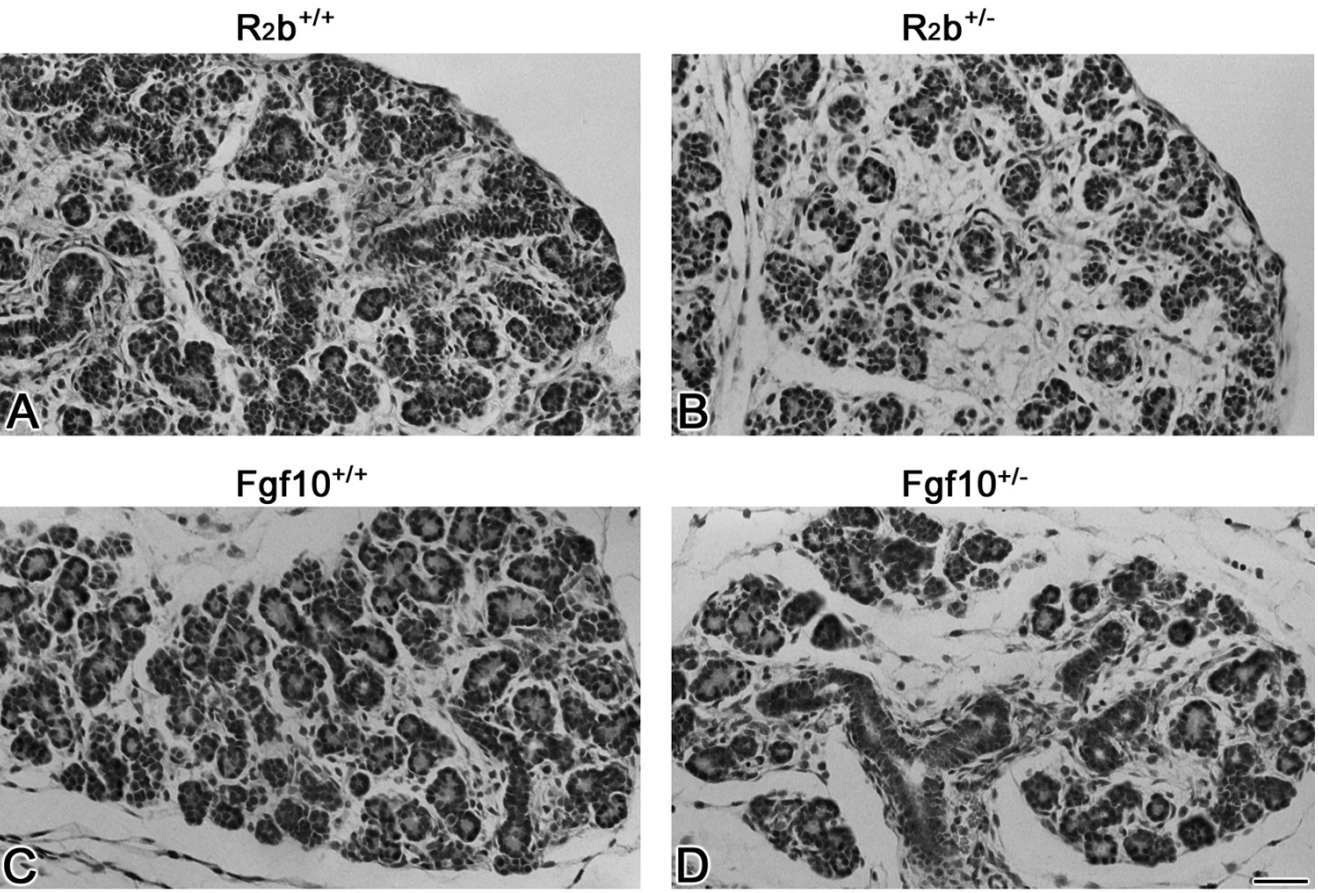
**Hypoplastic SMGs are seen in *Fgfr2b *and *Fgf10 *heterozygous mice. **A. Newborn WT *Fgfr2b*^+/+ ^SMG. B. Newborn *Fgfr2b*^+/- ^heterozygous SMG. C. Newborn WT *Fgf10*^+/+ ^SMG. D. Newborn *Fg10*^+/- ^heterozygous SMG. WT *Fgfr2b*^+/+ ^(A) and *Fgf10*^+/+ ^(C) mice exhibit *Late Terminal Bud *Stage SMGs consisting of ducts and terminal buds displaying distinct lumina. In contrast, fewer ducts and terminal buds are seen in *Fgfr2b*^+/- ^(B) and *Fg10*^+/- ^(D) heterozygous SMGs compared to WT littermates. Bar, 50 μm.

To delineate the role of FGF10/FGFR2b signaling during embryonic SMG development, we evaluated the SMG phenotype in *Fgf10 *and *Fgfr2b *null and heterozygous mutant mice. The E12.5 normal SMG appears as an elongated solid cord of epithelium terminating in an end-bulb (i.e., *Initial Bud *Stage) (Fig. 1A). By contrast, the E12.5 *Fgfr2b *null SMG is severely hypoplastic (compare Fig. 1B to 1A), displaying an extremely small initial bud similar to the earliest *Initial Bud *Stage [1,2]. A similar phenotype is seen in *Fgf10 *null mice (compare Fig. 1C to 1A, B). Normally by E13.5, epithelial proliferation results in SMG primordia with end-bulbs characterized by several branches; SMGs from both *Fgfr2b *and *Fgf10 *null mice are entirely absent (compare Fig. 2B, C to 2A). On the other hand, *Fgfr2b*^+/- ^and *Fgf10*^+/- ^heterozygosity results in SMG branching hypoplasia compared to WT glands, with fewer ducts and terminal buds being seen in heterozygous mutant SMGs than in WT SMG (compare Fig. 3B to 3A, D to 3C). Taken together, our data indicate that FGF10/FGFR2b signaling plays an essential, dose-dependent role during *in vivo *embryonic SMG branching morphogenesis and histodifferentation, but *not *earliest initial bud formation. Thus, the pathology in null mice is aplasia, not agenesis.

### Enhanced FGF10/FGFR2b signaling *in vitro *induces embryonic SMG branching morphogenesis and epithelial cell proliferation

**Figure 4:**
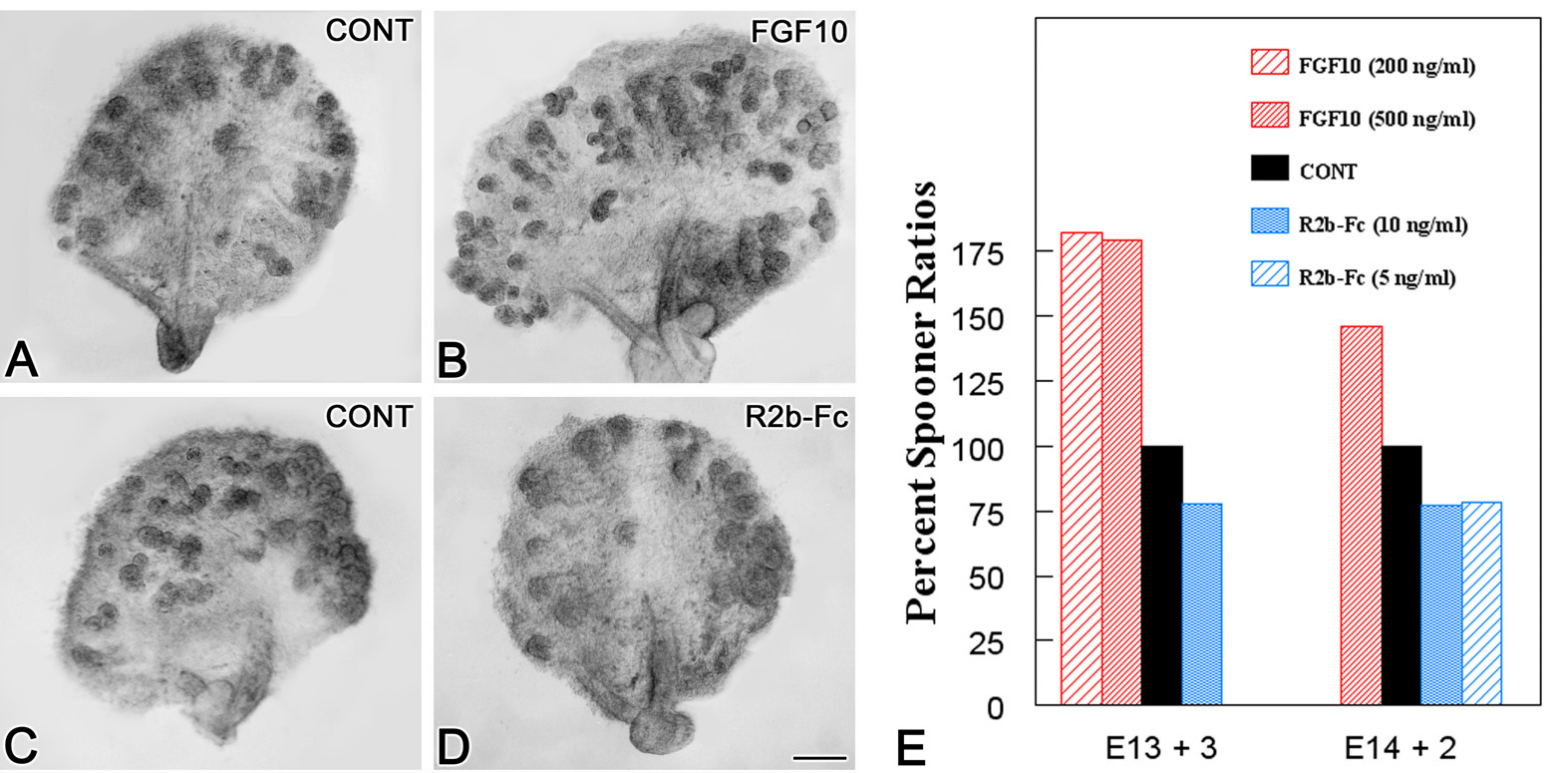
**Enhanced or abrogated FGF10/FGFR2b signaling modulates embryonic SMG branching morphogenesis *in vitro***. A-B. Enhanced signaling. Paired E13 SMG primordia were cultured for 3 days in the absence (A) or presence (B) of 500 ng/ml FGF10 peptide supplementation. FGF10 induced a significant increase in branching compared to control (CONT). C-D. Abrogated signaling. Paired E13 SMG primordia were cultured for 3 days in 10 ng/ml IgG-Fc (C) or FGFR2b-Fc chimera (D). FGFR2b-Fc chimera treated explant exhibits a significant decrease in branching compared to IgG-Fc control. Bar, 30 μm. E. Comparison of mean Spooner ratios in E13 + 3 and E14 + 2 explants. Exogenous FGF10 peptide supplementation induced a significant 81% increase in E13 +3 explants [200 ng/ml (P < 0.01); 500 ng/ml (P < 0.001)] and a significant 46% increase in E 14 + 2 explants (P < 0.0001) compared to controls. FGFR2b-Fc chimera-mediated interruption of E13 + 3 and E 14 + 2 SMGs significantly reduced branching morphogenesis by 22% [E13 +3: 10 ng/ml (P < 0.01); E 14 + 2: 5 ng/ml (P > 0.0001); 10 ng/ml (P < 0.01)] compared to controls.

To further delineate the role of FGF10/FGFR2b signaling during embryonic SMG development, we used our well-defined organ culture system to analyze the effect of enhanced FGF10/FGFR2b signaling on embryonic SMG branching morphogenesis. Paired E13 (*Initial Bud *Stage) or E14 (*Pseudoglandular *Stage) SMG primordia were cultured for up to 3 days in the presence or absence of FGF10 peptide (200 ng/ml or 500 ng/ml). Since a notable difference in SMG branch number is usually seen among littermates, we compared the number of terminal buds in right and left glands (treated and control) from each embryo. Spooner ratios (end bud number/initial bud number) were determined for each explant, the data were then arcsin transformed, and the mean ratios compared by paired t-test. FGF10 supplementation induced a significant increase in branching morphogenesis (Fig. 4A, B, E): 81% for E13 + 3 [200 ng/ml (P < 0.01); 500 ng/ml (P < 0.001)] and 46% for E14 + 2 [500 ng/ml (P < 0.0001)].

**Figure 5:**
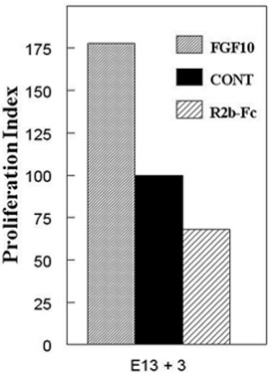
**Enhanced or abrogated FGF10/FGFR2b signaling modulates cell proliferation**. The cell proliferation index of E13 + 3 explants was calculated as the number of PCNA positive epithelial cells/total epithelial cells. Exogenous FGF10 peptide induced a significant 78% (P < 0.0001) increase, and FGFR2b-Fc chimera resulted in a significant 32% (P < 0.001) decrease, in cell proliferation compared to control.

Since cell proliferation is not required for early embryonic SMG epithelial branching [29], we determined if this FGF10-induced increase in branching is due to increased epithelial cell proliferation. We cultured E13+3 SMG primordia in the presence or absence of 500 ng/ml FGF10 peptide and calculated the epithelial cell proliferation index (the number PCNA-positive cells/total number of cells). Exogenous FGF10 induced a significant 78% (P < 0.0001) increase in epithelial cell proliferation compared to control (Fig. 5). Our results are similar to enhanced pancreatic epithelial cell proliferation and pancreatic hyperplasia in transgenic mice with persistent *Fgf10 *expression in developing pancreatic epithelia [30].

### Abrogated FGF10/FGFR2b signaling *in vitro *decreases embryonic SMG branching morphogenesis and cell proliferation

We interrupted FGF10/FGFR2b signaling *in vitro *by adding exogenous soluble FGFR2b-Fc chimera to the culture medium to competitively bind endogenous FGFR2b ligands. This exogenous receptor/ligand binding methodology has been successfully used to interrupt FGFR2b, FGFR2c, FGFR1b and FGFR1c signaling *in vitro *[13,15,21]. This experiment was designed to provide an *in vitro *model of the *in vivo Fgf10*^+/- ^heterozygous mutant SMG phenotype (i.e. SMG hypoplasia). We conducted dose-response studies and determined the concentration of FGFR2b-Fc chimera which induces a clear biologic effect of reduced branching morphogenesis, thereby creating an *in vitro *model of the *in vivo Fgf10 *mutant heterozygote. Paired E13+3 or E14+2 SMG primordia were cultured in IgG-Fc (5 ng/ml or 10 ng/ml) or FGFR2b-Fc chimera (5 ng/ml or 10 ng/ml) and Spooner ratios were determined as described above. E13 + 3 FGFR2b-Fc-treated explants exhibit a significant 22% [10 ng/ml (P < 0.01)] decrease in branching morphogenesis compared to controls (Figs. 4C, D, E); a similar 22% reduction [5 ng/ml (P < 0.001); 10 ng/ml (P < 0.01)] was also seen in E14+2-treated explants (Figs. 4E). These results mimic the *in vivo *heterozygous mutants reported above.

Since exogenous FGF10 supplementation *in vitro *induced a significant increase in cell proliferation, we then evaluated the epithelial cell proliferation index in E13 +3 SMG primordia with or without abrogated FGF10/FGFR2b signaling. A significant 32% (P < 0.001) decrease in cell proliferation was seen in the presence of 10 ng/ml FGFR2b-Fc chimera compared to control (Fig. 5). Reduction in cell proliferation with FGFR2b-Fc treatment *in vitro *was also reported by Steinberg et al., [21]. These results indicate that FGF10/FGFR2b signaling modulates embryonic SMG epithelial cell proliferation *and *branching morphogenesis.

### Relationship between FGFR2b and FGFR2c signaling pathways during SMG development

**Figure 6:**
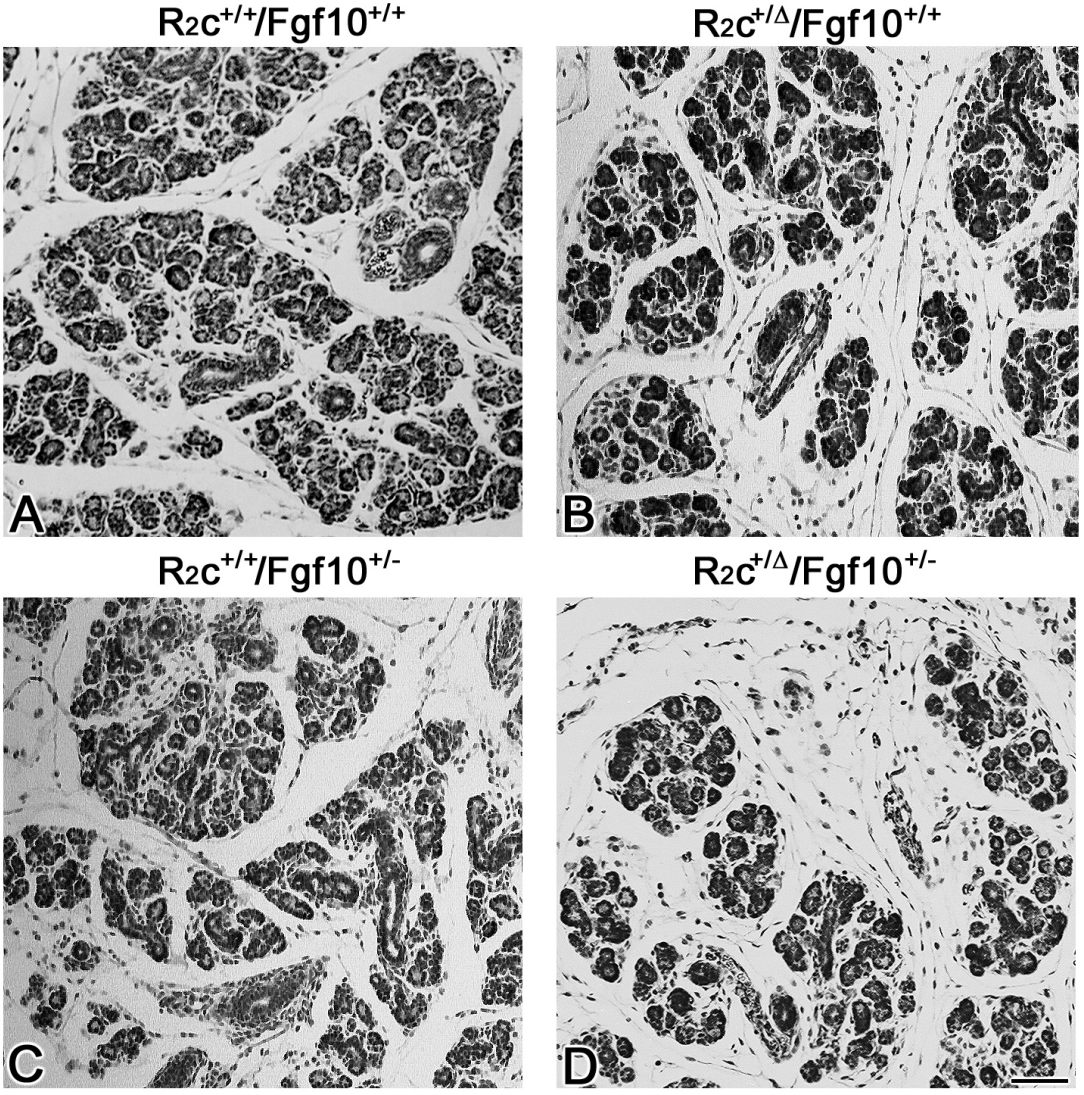
***Fgfr2c*^+/*Δ*^/*Fgf10*^+/- ^double heterozygous mutant SMG is severely hypoplastic**. A. Newborn *R2c*^+/+^*/Fgf10*^+/+ ^WT SMG. B. Newborn R*2c*^+/*Δ*^*/Fgf10*^+/+ ^(*Fgfr2c*^+/*Δ*^/*Fgf10*^+/+^) single heterozygous mutant SMG. C. Newborn *R2c*^+/+^/*Fgf10*^+/-^(*Fgfr2c*^+/+^/*Fgf10*^+/-^) single heterozygous mutant SMG. D. Newborn R*2c*^+/*Δ*^*/Fgf10*^+/-^(*Fgfr2c*^+/*Δ*^*/Fgf10*^+/-^) double heterozygous mutant SMG. The *Fgfr2c*^+/*Δ*^*/Fgf10*^+/+ ^(B) and *Fgfr2c*^+/+^/* Fgf10*^+/- ^(C) single heterozygous SMGs have fewer ducts and terminal buds than seen in WT littermates (A). A much smaller, severely hypoplastic gland is seen in *Fgfr2c*^+/*Δ*^*/Fgf10*^+/- ^double heterozygous mice (D) compared to *Fgfr2c*^+/*Δ*^*/Fgf10*^+/+ ^(B) and *Fgfr2c*^+/+^/*Fgf10*^+/- ^(C) littermates. Bar, 50 μm.

Our previous observation of hypoplastic SMGs in *Fgfr2c*^+/*Δ *^deficient mice suggested that FGFR2c signaling is necessary for embryonic SMG branching morphogenesis and histodifferentation [2]. The functional importance of this signaling pathway was confirmed *in vitro*; reduction of FGFR2c signaling *in vitro *resulted in a significant dose-dependent decrease in branching morphogenesis [13]. In addition, FGF8, a major FGFR2c ligand, has been shown to play an essential, nonredundant role during embryonic SMG development [13]. Like the hypoplastic SMG phenotype of *Fgfr2c *deficient mice, hypoplastic glands are seen in *Fgf8 *hypomorphic mice [13]. Importantly, SMG aplasia is seen in *Fgf8 *tissue-specific conditional mutant mice. To investigate the relationship between the FGFR2b and FGFR2c signal transduction pathways during *in vivo *SMG development, we evaluated the SMG phenotype in mice which are heterozygous for both the *Fgf10 *and *Fgfr2c *genes (i.e. *Fgfr2c*^+/*Δ*^*/Fgf10*^+/- ^mutants). Mutant mice heterozygous for either *Fgfr2c *(*Fgfr2c*^+/*Δ*^*/Fgf10*^+/+^) or *Fgf10 *(*Fgfr2c*^+/+^*/Fgf10*^+/-^) exhibit hypoplastic SMGs compared to WT (*Fgfr2c*^+/+^*/Fgf10*^+/+^) littermates (compare Fig. 6B, C to 6A). In contrast, *Fgfr2c*^+/*Δ*^*/Fgf10*^+/- ^double heterozygous mutant SMGs are smaller and exhibit fewer ducts and terminal buds than seen in single heterozygous *Fgfr2c*^+/*Δ*^*/Fgf10*^+/+ ^or *Fgfr2c*^+/+^*/Fgf10*^+/- ^mutant glands (compare Fig. 6D to 4B, C). These results suggest that, during embryonic SMG development, the FGFR2b and FGFR2c signal transduction pathways each induce at least some downstream targets that are idiosyncratic and not coincident.

### FGF8 rescues SMGs with interrupted FGF10/FGFR2b *in vitro*

**Figure 7:**
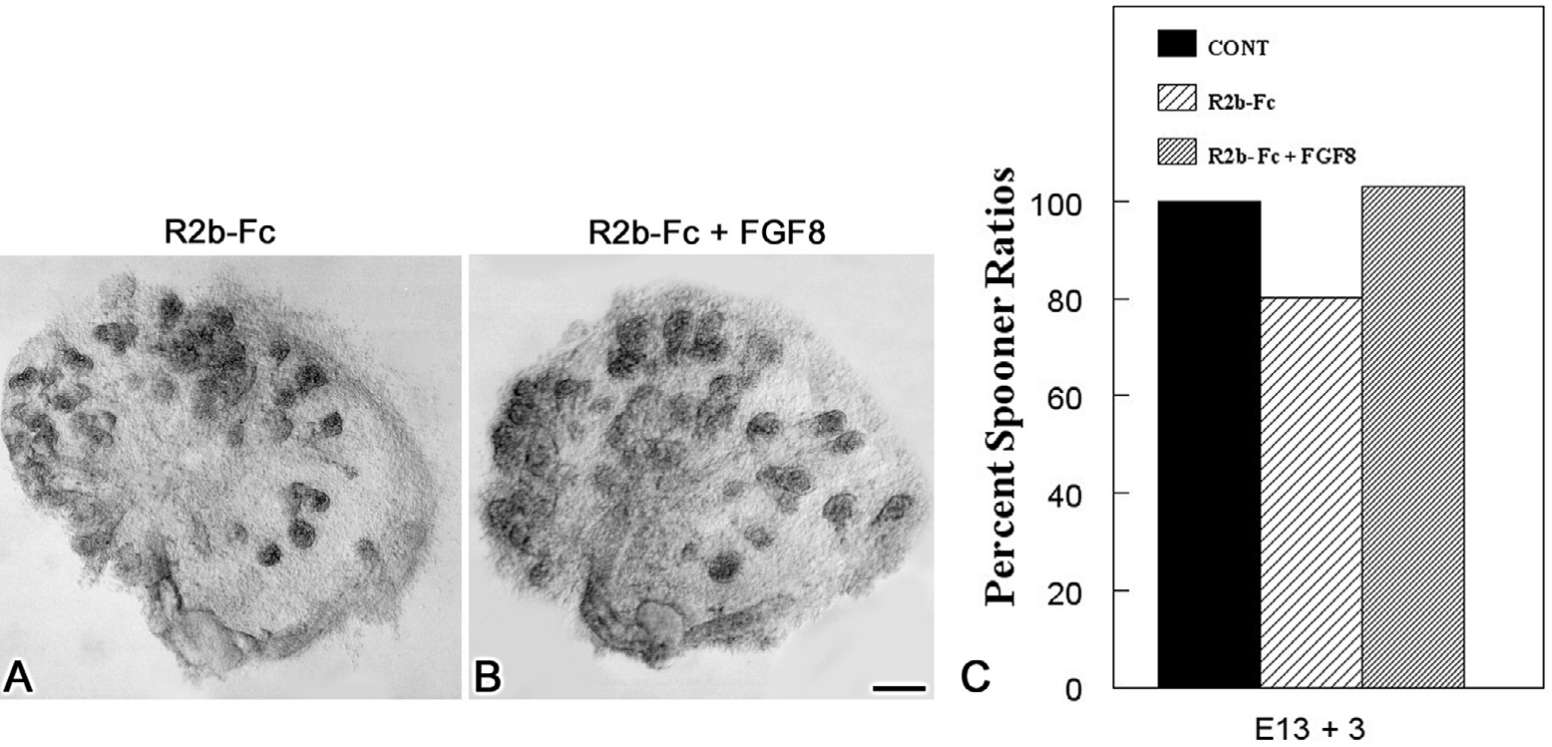
**Exogenous FGF8 supplementation *in vitro *rescues SMG branching morphogenesis. **Paired E13 embryonic SMGs were preincubated for 3 hrs in 10 ng/ml FGFR2b-Fc chimera and then cultured for a total of 3 days with/without 500 ng/ml FGF8 peptide. A. E13+3 10 ng/ml FGFR2b-Fc-treated explant. B. E13+3 FGFR2b-Fc chimera + FGF8-treated explant. Bar, 30 μm. C. Comparison of Spooner ratios. A significant 20% (P < 0.001) decrease in branching morphogenesis with FGFR2b-Fc chimera abrogation was seen compared to IgG-Fc control. Exogenous FGF8 supplementation induced a significant 24% (P < 0.02) increase in branching to completely restore branching to the level seen in control.

Given that FGF8-mediated signaling plays an essential and unique role during SMG development [13], as well as our observation in *Fgfr2c*^+/*Δ*^*/Fgf10*^+/- ^double heterozygous mutant mice discussed above, we postulated that exogenous FGF8 peptide would rescue the abnormal SMG phenotype associated with abrogated FGFR2b signaling *in vitro*. To determine if exogenous FGF8 peptide supplementation could restore branching morphogenesis to normal, we interrupted SMG morphogenesis *in vitro *with FGFR2b-Fc chimera and attempted to "rescue" these explants and restore branching to the level seen in controls. We cultured paired E13 + 3 SMGs in 10 ng/ml FGFR2b-Fc chimera for an initial period of 3 hours and then in FGFR2b-Fc + 500 ng/ml FGF8 peptide or FGFR2b-Fc alone for a total of 3 days (Fig. 7A, B). A second experiment to confirm that exogenous FGFR2b-Fc chimera decreased SMG branching morphogenesis consisted of paired E13 + 3 cultured in the presence of 10 ng/ml FGFR2b-Fc or IgG-Fc. In this set of experiments, FGFR2b-Fc-treated explants exhibit a significant 20% (P < 0.001) decrease in branching compared to IgG-Fc controls (Fig. 7C). Addition of exogenous FGF8 peptide induced a significant 24% (P < 0.02) increase in branching compared to FGFR2b-Fc treatment alone, thus completely restoring branching morphogenesis to the level seen in controls (Fig. 7C).

## Discussion

The FGF family of growth factors is critical to normal embryogenesis, regulating cell proliferation, survival and apoptosis [8]. Analyses of *Fgfr2b *and *Fgf10 *mutant and null mice clearly demonstrate that the FGF10/FGFR2b signal transduction pathway is essential for the development of branching organs, including the lung, mammary gland, lacrimal gland, pancreas, thyroid gland and salivary gland [23-28,31-33]. Although SMGs were absent from E14.5 or older *Fgfr2b *null mice and newborn *Fgf10 *null mice [22-25], the presence of an initial SMG bud in E12.5 *Fgf10 *and *Fgfr2b *null embryos (Fig. 1) indicates that this is a true aplasia, and not agenesis. Moreover, the absence of SMGs in E13.5 and older *Fgfr2b*^-/- ^and *Fgf10*^-/- ^mutants confirms that FGF10/FGFR2b signaling is essential for earliest initial epithelial branching and subsequent *Pseudoglandular *Stage and older SMG morphogenesis, but not earliest initial bud formation.

The observed initial SMG bud formation and subsequent aplasia is consistent with the pathogenesis seen in other organs, including the *Fgfr2b *null mammary gland [26], but differs from that seen in lung and pancreas [24,31,34]. Interestingly, Bellusci and colleagues [26] also detected genotype-specific phenotypic differences in mammary bud formation. A transient single initial mammary gland bud (bud 4) seen in E11.5 *Fgfr2b*^-/- ^mice is absent in E12.5 mice whereas bud 4 is maintained (but does not branch) in *Fgf10*^-/- ^mice. This result suggests that FGFR2b signaling is essential to maintain bud 4 and to induce the other mammary placodes whereas another FGFR2b ligand (probably FGF7) acts redundantly with FGF10 to maintain the mammary gland placode. Similarly, differences in pancreatic development were observed between *Fgf10 *and *Fgfr2b *null mice [31,35]. Taken together, these results clearly indicate that *Fgfr2b *and *Fgf10 *null mice demonstrate tissue-specific differences in affected organs.

Although *Fgf10*^+/- ^heterozygous mice were described as being normal [24,34], lacrimal, parotid and submandibular gland aplasia or hypoplasia were recently reported in adult *Fgf10*^+/- ^mice and in ALSG patients with *FGF10 *heterozygous mutations [28]. To determine if *Fgfr2b *and *Fgf10 *gene dosage plays an important role during embryonic SMG development, we evaluated newborn *Fgfr2b*^+/- ^and *Fgf10*^+/- ^and found SMG hypoplasia in both (Fig. 3). This is the first report of organ abnormality in *Fgfr2b *heterozygous mice. Our data indicate that SMG development is *Fgf10 *and *Fgfr2b *dose-dependent. Homozygous null mutants exhibit SMG aplasia while heterozygotes exhibit SMG hypoplasia. Moreover, the observations of normal lungs, livers, and limbs [23-25,28,34], but abnormal SMG phenotypes [28], in *Fgfr2b*^+/- ^and *Fgf10*^+/- ^mutant mice provides additional evidence of FGF10/FGFR2b tissue-specificity.

### Relationship between FGF10/FGFR2b and FGF8/FGFR2c signaling pathways

It is critical to remember that FGF10 binding to FGFR2b is part of a much larger genetic network. Organogenesis is the programmed expression of regulatory genes coupled to downstream structural genes and epigenetic events. Specific signaling pathways are parallel and largely functionally redundant; that is, several pathways differentially and combinatorially compensate for the dysfunction of a given individual pathway. There are some pathways, however, that have unique and nonredundant functions. One has always to ask two key questions: Is our pathway of interest functionally redundant or nonredundant? Will a broadly related, not independent, pathway compensate for the dysfunction of our pathway of interest?

The observation of SMG aplasia in Fg*f10 *and *Fgfr2b *null mice indicates that the functional presence of other endogenous FGF/FGFR pathways (e.g., FGF8/FGFR2c, FGF/FGFR1) or other signaling pathways (e.g., TGF-α/EGF/EGFR, Eda/Edar; IGF-II/IGF-IR) could not prevent complete death of embryonic SMG cells in *Fgf10*^-/- ^and *Fgfr2b*^-/- ^mice. Interestingly, although SMG aplasia was also seen in *Fgf8 *(*Fgf8*^*C*/*N*^*;AP2α*^*IRESCre*/+^) conditional mutant mice in which *Fgf8 *expression was ablated from first branchial arch epithelium, a small initial SMG bud is still seen in E15.5 embryos [13]. By contrast, the *Fgf10*and *Fgfr2b *null SMG bud is much more transient, being seen in E12.5 and absent in E13.5 mice (present study). This suggests that the FGF10/FGFR2b and FGF8/FGFR2c signaling pathways are both essential for branching and that the FGF10/FGFR2b signal is necessary for epithelial bud maintenance at earlier stages than the FGF8/FGFR2c signal.

The absence of SMGs in both *Fgf8*^*C*/*N*^*; AP2α*^*IRESCre*/+ ^conditional mutant [13] and *Fgf10*^-/- ^null mice (present study) suggests that FGF8 and FGF10 probably induce some of the same downstream targets, although through different receptors (FGF10/FGFR2b and FGF8/FGFR2c). However, the mere fact of ontogenic arrest and SMG aplasia, as well as temporal differences, in *Fgf8 *conditional mutants and *Fgf10 *and *Fgfr2b *null mice indicate that FGF10/FGFR2b and FGF8/FGFR2c signaling pathways induce broadly-related, but unique and nonredundant downstream cascades in SMGs that cannot be compensated by normal function of the other under physiologic conditions. Our observation of smaller and more severely hypoplastic SMGs in *Fgfr2c*^+/*Δ*^*/Fgf10*^+/- ^double heterozygous mice compared to either the *Fgfr2c*^+/*Δ *^or *Fgf10*^+/- ^single heterozygous mutants further supports this conclusion.

It is well established that FGF/FGFR signaling can simultaneously activate multiple signaling cascades (e.g., ERK/RAS/MAPK, P13K, and PLC-γ/PKC to mediate epithelial cell proliferation, survival and histodifferentiation [8,9,11]. Inhibition of ERK/RAS/MAPK or P13K signaling significantly reduced embryonic SMG branching morphogenesis *in vitro *[17-19,21], whereas inhibition of PKC modestly increased morphogenesis [18]. A recent study of FGFR2b signaling *in vitro *suggests that FGF10/FGFR2b requires ERK activation to mediate cell proliferation and branching whereas FGF7/FGFR2b requires both ERK and P13K activation [21].

Although the importance of ERK/RAS/MAPK, P13K and PKC signaling during embryonic SMG development *in vitro *has been demonstrated, it is presently unclear which signaling cascades downstream of the FGF10/FGFR2b and FGF8/FGFR2c pathways are essential for epithelial branching morphogenesis, proliferation and survival during *in vivo *SMG development. This is important because *in vitro *and *in vivo *results may not necessarily coincide, suggesting the effect of differing physiologic conditions. Recently, Steinberg et al. [21] demonstrated that, *in vitro*, once the gland is formed, inhibition of FGF10 does not inhibit branching. This is the precise opposite of that seen in both the *Fgf10*^-/- ^null mice and the *Fgf10*^+/- ^heterozygotes *in vivo *(Fig. 1C, 2C, 3C), as well as *FGF10 *heterozygous mutant individuals with ALSG syndrome [28].

*Fgf10 *(*Fgf10*^-/-^) or *Fgfr2b *(*Fgfr2b*^-/-^) loss of function is ultimately epistatic to each other and to the epigenome under normal physiologic conditions (i.e. no other gene mutations nor untoward environments), the very reason they are critical to SMG morphogenesis. However, since the epistasis associated with declining *Fgf10 *or *Fgfr2b *function is a nonlinear emergent property of the complete functional epigenotype, it can be manipulated *in vitro *in the manner reported here. Exogenous FGF8 peptide can completely rescue and restore to normal the abnormal phenotype seen with abrogated FGF10/FGFR2 signaling *in vitro *(Fig. 7). This is not surprising since FGF8 has the ability to simultaneously activate similar, as well as unique and ligand-specific, intracellular cascades which control proliferation, survival, and differentiation. Rescue experiments have always to be only a proof of this principle, not a mimic of the *in vivo *condition. After all, *Fgf10*^-/- ^and *Fgfr2b*^-/- ^mutant SMGs are not rescued *in vivo*, the very essence of epistatic mutations. What remains to be determined is the *in vivo *functional relationship between the FGF10/FGFR2b signal transduction pathway and other key downstream signaling pathways, and how these pathways are integrated during embryonic SMG development to compose the functional epigenome.

## Conclusion

Our results indicate that FGF10/FGFR2b signaling is essential for the SMG epithelial branching and histodifferentiation, but not earliest initial bud formation. The functional presence of other endogenous FGF pathways or other signaling pathways could not prevent complete death of embryonic SMG cells in *Fgf10 *and *Fgfr2b *null mice. Moreover, our analysis of *Fgfr2c*^+/*Δ*^*/Fgf10*^+/- ^double heterozygous mice indicates that FGF10/FGFR2b and FGF8/FGFR2c signaling pathways induce broadly-related, but unique and nonredundant downstream cascades in SMGs. What remains to be determined is the *in vivo *functional relationship between the FGF10/FGFR2b signal transduction pathway and other key downstream signaling pathways, and how these pathways are integrated during embryonic SMG development to compose the functional epigenome.

## Methods

### *Fgf10 *and *Fgfr2b *mutant mice

*Fgf10 *and *Fgfr2b *mutant mice were generated on a C57Bl/6 background and genotyped by RT-PCR as previously described [23,26,34]. Wildtype mice (WT) (littermates or otherwise) were used as controls. WT (E11.5-E18.5), *Fgf10*^-/- ^(E11.5-5-E14.5) and *Fgfr2b*^-/- ^(E11.5-E15.5) embryos were collected. For analysis of heterozygous SMGs, WT and heterozygous mice were mated and newborn *Fgf10*^+/-^, *Fgfr2b*^+/- ^and WT littermates were collected and their genotypes confirmed by RT-PCR. The embryos and newborn heads were fixed in 4% paraformaldehyde in PBS, and stored in 70% ethanol until further processing. E11.5-E14.5 heads and E15 and older SMGs were processed, embedded in paraplast and serial coronal sections were stained with hematoxylin and eosin as previously described [1]. A minimum of 3 SMGs per age per genotype were analyzed.

### Generation of *Fgfr2c*^+/*Δ*^/*Fgf10 *+/- mice

Due to their neonatal lethality, *Fgfr2-IIIc*^+/*Δ *^mice are routinely generated by crossing males in which a copy of FgfR2-exon 9 (IIIc) has been flanked by loxP sites [36], with females carrying a PGK-Cre transgene [37]. However, to obtain the *Fgfr2-IIIc *^+/*Δ*^*;Fgf-10 *^+/- ^allele, we used PGK-Cre females in to which we had previously introduced a heterozygous *Fgf10 *null allele (*Fgf10 *^+/-^) [27]. For simplicity, the *Fgfr2-IIIc *^+/*Δ*^*;Fgf-10 *^+/- ^is called *Fgfr2c *^+/*Δ*^*/Fgf-10 *^+/-^. This cross resulted in the recovery of the following genotypes in the correct Mendelian ratios: *Fgfr2c *^+/+^*/ Fgf10 *^+/+ ^(WT); *Fgfr2c *^+/+^*/ Fgf10 *^+/-^, *Fgfr2c *^+/*Δ*^*/Fgf-10 *^+/+ ^and *Fgfr2c *^+/*Δ*^*/Fgf-10 *^+/- ^[36]. Lines were maintained and crosses were performed in a C57/black 6 background. E17-newborn WT and mutant mice were generated, SMGs were fixed and processed as described above, and their genotypes confirmed by RT-PCR as previously described [27,36]. All animal studies were conducted with the approval of the appropriate committees regulating animal research.

### Culture system

Timed-pregnant females [C57Bl/10 (B10.A)] were sacrificed on day 13 and day 14 of gestation, and embryos were dissected in cold PBS and staged according to Theiler [38]. E13 (*Initial Bud *Stage) and E14 (*Pseudoglandular *Stage) SMG primordia were cultured for up to 3 days using a modified Trowell method as previously described [6]. The medium consisted of BGJb (Life Technologies, Rockville, MD) supplemented with 1% BSA, 0.5 mg ascorbic acid/ml and 50 units penicillin/streptomycin (Life Technologies), pH 7.2, and replicate cultures were changed every day. *Supplementation studies*: paired E13 and E14 SMG primordia were cultured in the absence or presence of exogenous FGF10 peptide (200 or 500 ng/ml, R and D Systems) for 3 days (E13 + 3) or 2 days (E14 + 2), respectively; controls consisted of enriched BGJb alone. Because a notable difference in SMG epithelial branch number is seen between embryos within a given litter and among litters, we calculated the Spooner branch ratios (end bud number/initial bud number) for each explant as previously described [13] and compared the Spooner branch ratios in right and left glands (treated and control) from each embryo. Mean Spooner ratios were determined, the data were arcsin transformed to insure normality and homoscedasticity, and compared by paired *t*-test for all embryos studied [39]. In this set of experiments, 4–6 explants per treatment were analyzed.

#### Interruption studies

We interrupted FGF10/FGFR2b signaling using soluble FGFR2b-Fc chimera (R & D Systems, Inc). This method has been successfully used to interrupt FGFR2b, FGFR2c, FGFR1b and FGFR1c signaling during embryonic SMG development [13,15,21]. We conducted dose-response studies in which we cultured paired E13 + 3 or E14 + 2 SMG primordia in the presence of soluble FGFR2b-Fc chimera (5 or10 ng/ml) or control IgG-Fc (5 or 10 ng/ml IgG-Fc; R & D Systems, Inc), analyzed Spooner ratios as described above, and determined the optimal FGFR2b-Fc chimera concentration which results in a hypoplastic gland similar to that seen in the *in vivo Fgf10 *mutant heterozygotes. In this set of experiments, 4–8 explants per treatment were analyzed. Based on this set of experiments, we used 10 ng/ml FGFR2b-Fc chimera in all subsequent interruption experiments.

### Cell proliferation assay

The cell proliferation index was determined as previously described [6]. Paired E13 + 3 were cultured in 10 ng/ml FGFR2b-Fc or 10 ng/ml IgG-Fc, fixed in 10% formalin, embedded in paraffin and serially-sectioned. The sections were stained with anti-PCNA for 1 hr using the Zymed mouse PCNA kit (South San Francisco, CA) and counterstained with hematoxylin as previously described [13]. The development time was adjusted according to experiment: 2–5 min for supplementation studies and 10–15 min for interruption studies. In these experiments, the cytoplasm appears blue and PCNA-positive cells appear brown. Three explants per group were analyzed. Control and treated explants were serially-sectioned and the midpoint of each explant identified. The section showing the explant's mid-point was selected, as well as the fourth section to the right and the fourth section to the left of the midpoint. This design insured that different buds were counted in each of the 3 sections/treatment. In each section, we photographed terminal bud clusters in the upper left and lower right at 400 × and counted a minimum of 3 buds/area.

Cell proliferation was quantitated as the ratio of PCNA-positive epithelial cells/total epithelial cells. The mean cell proliferation index was determined per section and the mean cell proliferation index of the three sections/explant was determined for each treatment. The data was arcsin transformed and the means ratios compared by t-test.

### Rescue experiment

Paired E13 SMG primordia were cultured in 10 ng/ml FGFR2b-Fc chimera for an initial period of 3 hrs and then each pair was cultured in FGFR2b-Fc or FGFR2b-Fc + 500 ng/ml FGF8 (R and D Systems, Inc.) for 3 days. This FGF8 peptide concentration was determined to induce a significant increase in branching morphogenesis (data not shown). A concurrent control experiment was conducted as an internal control to verify that FGFR2b-Fc chimera supplementation interrupted branching morphogenesis; these controls consisted of E13 primordia cultured in 10 ng/ml FGFR2b-Fc chimera or 10 ng/ml IgG-Fc for 3 days. The explants were collected and mean Spooner ratios determined and compared as described above. Four explants per treatment were analyzed.

## List of abbreviations

ALSG-aplasia of lacrimal and salivary gland

BSA-bovine serum albumen

CONT-control

FGF-Fibroblast growth factor

FGFR2b-FGF receptor 2-IIIb

FGFR2c-FGF receptor 2-IIIc

ERK-p44/p42 mitogen activating kinases (ERK-1/2)

MAPK-mitogen-activating protein kinases

P13K-phosphatidylinositol 3-kinase

PBS-phosphate-buffered saline

PKC-protein kinase C

PLC-γ-phospholipase C γ1

R2b^-/-^-Fgfr2b^-/-^

R2c-Fgfr2c

RT-PCR-reverse transcriptase-polymerase chain reaction

SMG-submandibular salivary gland

WT-wildtype

## Authors' contributions

TJ designed and coordinated the study, was involved in aspects of all experiments, and drafted the manuscript. GA prepared the histological sections for this study and performed some of the morphology and cell proliferation experiments. DW participated in morphological analyses and culture experiments and generated all figures. FGS generated the *Fgf10 *and *Fgfr2b *mutant mice and harvested and genotyped the embryos. SB assisted in the analyses of mutant mouse histopathology. MKH generated the *Fgfr2c*^+/*Δ*^*/Fgf10 *^+/- ^single and double mutant mice, harvested and genotyped these embryos, and assisted in the analysis of their histopatholgy. MM participated in the design and coordination of this study, assisted in the analysis of histopathology, performed the statistical analysis, and helped draft the manuscript. All authors read and approved the final manuscript.
